# Early life exposures shape the CD4^+^ T cell transcriptome, influencing proliferation, differentiation, and mitochondrial dynamics later in life

**DOI:** 10.1038/s41598-019-47866-2

**Published:** 2019-08-07

**Authors:** Catherine G. Burke, Jason R. Myers, Lisbeth A. Boule, Christina M. Post, Paul S. Brookes, B. Paige Lawrence

**Affiliations:** 1 Department of Microbiology & Immunology, University of Rochester School of Medicine & Dentistry, Rochester, NY 14624 USA; 2 Genomics Research Center, University of Rochester School of Medicine & Dentistry, Rochester, NY 14624 USA; 3 Department of Environmental Medicine, University of Rochester School of Medicine & Dentistry, Rochester, NY 14624 USA; 4 Department of Anesthesiology, University of Rochester School of Medicine & Dentistry, Rochester, NY 14624 USA

**Keywords:** Lymphocytes, Adaptive immunity, Cellular immunity

## Abstract

Early life environmental exposures drive lasting changes to the function of the immune system and can contribute to disease later in life. One of the ways environmental factors act is through cellular receptors. The aryl hydrocarbon receptor (AHR) is expressed by immune cells and binds numerous xenobiotics. Early life exposure to chemicals that bind the AHR impairs CD4^+^ T cell responses to influenza A virus (IAV) infection in adulthood. However, the cellular mechanisms that underlie these durable changes remain poorly defined. Transcriptomic profiling of sorted CD4^+^ T cells identified changes in genes involved in proliferation, differentiation, and metabolic pathways were associated with triggering AHR during development. Functional bioassays confirmed that CD4^+^ T cells from infected developmentally exposed offspring exhibit reduced proliferation, differentiation, and cellular metabolism. Thus, developmental AHR activation shapes T cell responsive capacity later in life by affecting integrated cellular pathways, which collectively alter responses later in life. Given that coordinated shifts in T cell metabolism are essential for T cell responses to numerous challenges, and that humans are constantly exposed to many different types of AHR ligands, this has far-reaching implications for how AHR signaling, particularly during development, durably influences T cell mediated immune responses across the lifespan.

## Introduction

Every day humans are exposed to myriad environmental factors through the air we breathe, the food and beverages we consume, products we interact with, and many activities or jobs we undertake. While some of these exposures are beneficial, others are detrimental to human health. Indeed, 25% of, or over 12.6 million, deaths every year are attributable to environmental factors, which means that these deaths are avoidable^1^. In particular, environmental exposures that occur during developmentally sensitive periods, such as in the womb and shortly after birth, are associated with an increased risk of developing cardiovascular disease, obesity, cancer, and diabetes later in life^2,3^. Exposures during development are also associated with changes to the function of the immune system^4^. For instance, several epidemiological studies have linked early life exposure to pollutants that act through the aryl hydrocarbon receptor (AHR) with increased wheezing, decreased antibody responses to vaccination, and increased incidence of ear and respiratory tract infections later in life^5–12^. Despite these associations, the cellular mechanisms that drive altered immune function are poorly understood.

The major function of the immune system is to protect against infectious agents by detecting and destroying the pathogen and infected cells. Thus, a properly functioning immune system is critical to our ability to fight infections. Yet, respiratory infections are among the top five most common causes of death worldwide, regardless of income^1,13^. Among common respiratory pathogens are influenza viruses, which infect at least 1 billion people annually, resulting in several million hospitalizations, and at least ½ million deaths^14,15^. Successfully fighting influenza virus requires the activation, differentiation, and function of several immune cell types. Among these, CD4^+^ T cells are key to defending against influenza virus infection^16^. The two most abundant CD4^+^ T helper subsets during influenza A virus (IAV) infection are Th1 and T follicular helper (Tfh) cells. They produce antiviral cytokines, aid B cell activation and antibody class switching, and promote and maintain immunological memory^17,18^. The presence of another CD4^+^ T cell subset, Th17 cells, correlates with decreased mortality, although their precise role thwarting IAV infection remains uncertain^19,20^. Direct exposure to AHR ligands influences CD4^+^ T cell responses, including Th1 and Tfh cells, during IAV infection^21^. Recent evidence shows developmental AHR activation also influences CD4^+^ T cell responses. Specifically, developmental exposure to the prototype AHR ligand 2,3,7,8-tetrachlorodibenzo-*p*-dioxin (TCDD) causes lasting changes in the CD4^+^ T cell response to IAV infection in mice, which is partly due to direct effects on the CD4^+^ T cell lineage^22^. Despite the fact that developmental exposure alters CD4^+^ T cell responses during an immune challenge, naïve adult offspring do not exhibit any overt changes to immune organ cellularity, including the number or distribution of naïve CD4^+^ T cells^22–25^. These observations suggest that developmental AHR activation results in cryptic changes to the function of CD4^+^ T cells that are only revealed when CD4^+^ T cells are challenged to respond.

The response of CD4^+^ T cells is regulated by the coordinated action of many different signaling pathways. To pinpoint candidate functions and pathways that are durably affected by developmental AHR activation, we combined fluorescence activated cell sorting and transcriptome analysis. Specifically, after IAV infection, we separated resting and responding CD4^+^ T cells and performed unbiased transcriptome profiling to identify differentially expressed genes in adult offspring that were and were not exposed to TCDD during development. Then, we used functional bioassays to examine the proliferation, differentiation, and metabolic potential of CD4^+^ T cells following activation of the AHR during development. Identifying cellular functions that underlie intrinsic changes to CD4^+^ T cells following exposure to an AHR ligand during early life helps us understand how environmental exposures can cause long lasting influence on immune function.

## Results

### Developmental AHR activation changes gene expression in CD4^+^ T cells

**Figure 1:**
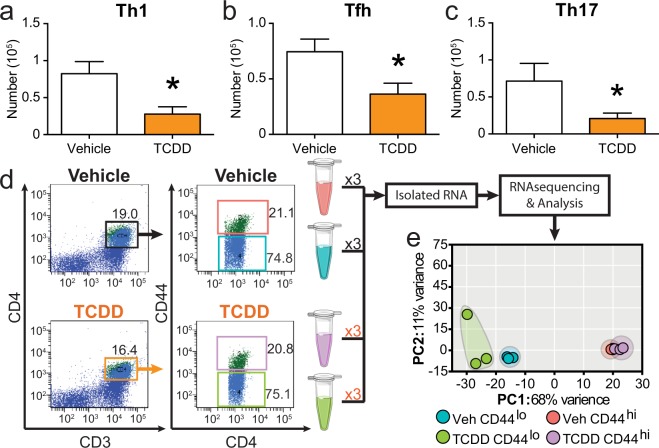
Experimental design to identify cellular pathways driving impaired CD4^+^ T cell responses following developmental AHR activation. C57Bl/6 mice were developmentally exposed to vehicle (Veh) or TCDD on gestational days 0, 7, 14, and post-natal day 2. At maturity (6–10 weeks), offspring were infected with influenza A virus (IAV). On day 9 post infection, MLN cells were stained for flow cytometry. CD4^+^ T cell subsets were identified using lineage specific markers as follows: Th1 cells TBet^+^CD4^+^, Tfh cells CD44^hi^CXCR5^+^PD-1^+^CD4^+^, and Th17 cells RORγt^+^CD4^+^. Bar graphs show the number of (**a**) Th1, (**b**) Tfh, and (**c**) Th17. Nine days after infection, resting (CD44^lo^) and responding (CD44^hi^) CD4^+^ T cells from the MLN were purified by FACS, RNA isolated, and RNA-sequencing (RNA-seq) was performed. To provide sufficient material, CD4^+^ T cells were isolated from 8–9 offspring derived from 3–4 unique dams per sample prior to sorting. None of the 6 pools of cells contained cells from the same dams. (**d**) Representative FACS purification gating strategy shows initial gate on CD3^+^CD4^+^ cells (left plots), and gating to sort responding CD44^hi^ and resting CD44^lo^ CD4^+^ T cells (right plots). (**e**) Principle component analysis (PCA) was used to determine the extent of variability between each sample and sample groups. In bar graphs, values are mean ± SEM. Values on flow plots indicate the average of the samples used for RNA-seq. Data for bar graphs are from 6–10 female offspring per treatment group. All offspring were born to separate dams. Experiments, other than RNAseq, were repeated at least once with similar results. An * signifies a p-value ≤ 0.05 by Student’s t-test. For the RNA-seq experiment, n = 3 replicates per group. Graphic art is from Servier Medical Art templates (https://smart.servier.com) which are licensed under the Creative Commons Attribution 3.0 Unported License agreement (https://creativecommons.org/licenses/by/3.0/).

Adult offspring that were developmentally exposed to the AHR ligand TCDD have fewer Th1, Tfh, and Th17 cells upon infection with IAV (Fig. 1a–c). The activation and differentiation of naïve CD4^+^ T cells into functionally distinct conventional helper T cell subsets involves the integration of multiple signaling pathways. This complexity means that there are many different genes and cellular processes in CD4^+^ T cells that could be affected by AHR activation during development. To identify which cellular pathways were changed as a consequence of triggering AHR during development, we compared the transcriptome of CD4^+^ T cells from IAV infected adult mice that were or were not exposed to TCDD during development. Specifically, we used fluorescent activated cell sorting (FACS) to separate resting (CD44^lo^) and responding (CD44^hi^) CD4^+^ T cells, and performed high throughput RNA sequencing (RNASeq) using three experimental replicates (Fig. 1d). Principle component analysis revealed that samples most closely clustered with replicates from their own group (Fig. 1e). Additionally, the first principle component separated the groups by activation state regardless of developmental exposure, which indicates activation state accounts for the greatest variance in the data set.

**Figure 2:**
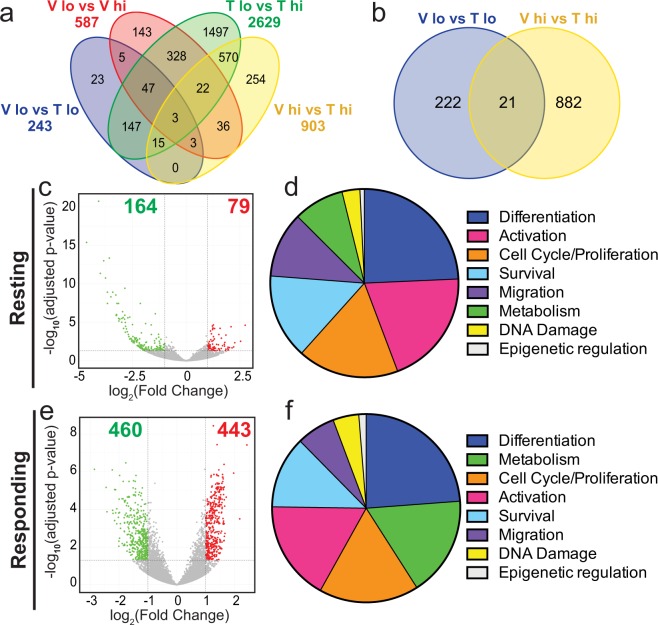
Developmental exposure to TCDD alters gene expression in CD4^+^ T cells following IAV infection. (**a**) 4-way and (**b**) 2-way Venn diagrams of differentially expressed genes (DEGs). Comparison groups are as follows: Blue- Vehicle CD44^lo^ vs. TCDD CD44^lo^; Red- Vehicle CD44^lo^ vs. Vehicle CD44^hi^; Green- TCDD CD44^lo^ vs. TCDD CD44^hi^; Yellow- Vehicle CD44^hi^ vs. TCDD CD44^hi^. (**c**,**e**) Volcano plots depict DEGs that are down-regulated (green dots) and up-regulated (red dots) by developmental exposure to TCDD in resting (CD44^lo^, **c**) and responding (CD44^hi^, **e**) CD4^+^ T cells compared to vehicle controls. (**d**,**f**) For these same comparisons, Ingenuity Pathways Analysis (IPA) was used. Predicted pathways were grouped by cellular function, and pie charts represent the number of pathways that were associated with differentiation, activation, cell cycle/proliferation, survival, migration, metabolism, DNA damage, or epigenetic regulation. The most represented cellular functions are listed in descending order in resting (CD44^lo^, **d**) and responding (CD44^hi^, **f**) CD4^+^ T cells.

Consistent with the distinctive cellular attributes of resting and responding T cells, there were overall changes in gene expression in CD44^lo^ versus CD44^hi^ CD4^+^ T cells in adult offspring from both developmental exposure groups (Fig. 2a). While some differentially expressed genes (DEGs) were shared between multiple comparisons, many were uniquely expressed within a specific comparison. For instance, in CD4^+^ T cells isolated from offspring of vehicle control-treated dams, there were 587 DEGs in resting (CD44^lo^) compared to responding (CD44^hi^) CD4^+^ T cells. Of these, 143 genes were unique to this comparison. The number of genes that were differentially expressed in CD44^lo^ versus CD44^hi^ CD4^+^ T cells from offspring of TCDD-treated dams (2629 genes) was 4.5 times greater than in the vehicle group. Of these 2629 DEGs, only 400 overlapped in resting versus responding CD4^+^ T cells across the two exposure groups. Notably, resting versus responding CD4^+^ T cells from mice developmentally exposed to TCDD had 1497 uniquely expressed DEGs. This reflects nearly 3 times more DEGs than the total number of DEGs (587 genes) in resting versus responding CD4^+^ T cells from offspring of control dams. This indicates that developmental exposure caused extensive and discrete gene changes, which are revealed when T cells become activated. Another informative comparison is the number of DEGs in CD4^+^ T cells that share the same cellular state. Consistent with their more quiescent state, there were 243 DEGs in resting CD4^+^ T cells, compared to 903 DEGs in responding CD4^+^ T cells. Moreover, there were only 21 DEGs were differentially expressed in both comparisons (Fig. 2b), which suggests developmentally induces changes in gene expression are dependent on activation state. Further, in resting CD4^+^ T cells, two-thirds of DEGs exhibited diminished expression (Fig. 2c), while the distribution of enhanced and decreased DEGs were in roughly equal proportions in responding CD4^+^ T cells (Fig. 2e). Collectively, these findings indicate that the bulk of the genes changed by developmental AHR activation depends on the activation state of the cell, and that the transcriptional profile of the T cells is rendered different by early life exposure.

To identify the major cellular functions represented by these DEGs, Ingenuity Pathway Analysis (IPA) was performed comparing resting (Fig. 2d) or responding CD4^+^ T cells (Fig. 2f) from offspring of vehicle and TCDD exposed dams. In resting CD4^+^ T cells, the top three cellular functions that were affected by developmental exposure were differentiation, activation, and cell cycle/proliferation (Fig. 2d). In responding cells, the top three cellular functions represented differentiation, metabolism, and proliferation (Fig. 2f). Thus, although activation state dictates which particular genes were dysregulated, developmental triggering of the AHR impacts similar cellular functions in CD4^+^ T cells regardless of their activation state. To empirically determine if CD4^+^ T cell proliferation, differentiation, and cellular metabolism were changed by developmental AHR activation we used functional bioassays.

### Activation of the AHR during development reduces CD4^+^ T cell proliferation

**Figure 3:**
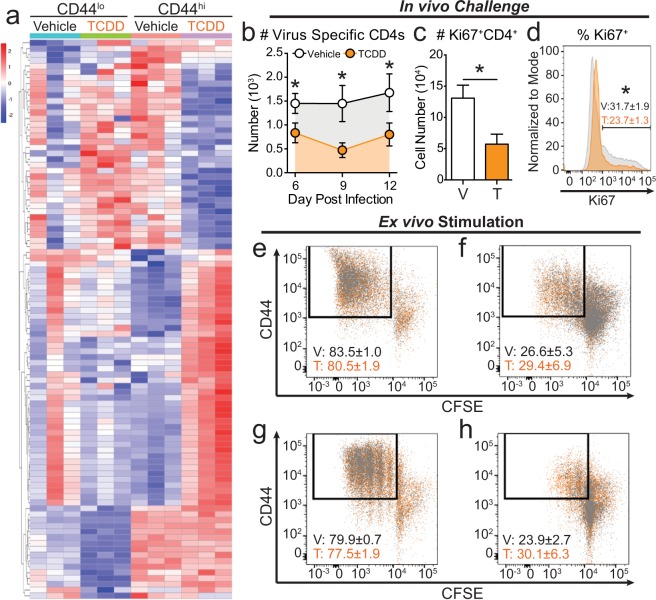
TCDD exposure during development impairs *in vivo* CD4^+^ T cell proliferation. (**a**) IPA predicted pathways involved in cellular proliferation. The heat map shows genes that are differentially expressed following developmental AHR activation in resting and responding CD4^+^ T cells. Genes were ordered using unsupervised clustering by row. See Supplemental Table 2 for gene list. (**b**–**d**) Adult offspring from Vehicle (V) and TCDD (T) exposed dams were infected with IAV. (**b**) Virus specific CD4^+^ T MLN cells were enumerated using flow cytometry on days 6, 9, and 12 post-infection using MHCII tetramers (I-A^b^/NP_311–325_). (**c**,**d**) Proliferating Ki67^+^CD4^+^ T cells were assessed on day 9 post-infection. Bar graph shows the (**c**) number in vehicle (white bar) and TCDD (orange bar) groups. The histogram shows the (**d**) percentage of CD4^+^ T cells that are Ki67^+^ in vehicle (grey histogram) and TCDD (orange histogram) mice. (**e**–**h**) CD4^+^ T cells were isolated from peripheral lymph nodes of naïve vehicle (grey dots) and TCDD (orange dots) developmentally exposed animals. Cells were stained with CFSE and stimulated in culture for (**e**,**f**) four or (**g**,**h**) three days with (**e**,**g**) 5 μg/mL or (*F*,*H*) 2.5 μg/mL of α-CD3/α-CD28. All values are mean ± SEM. At each point in time, all offspring within the same group were from a separate dam, and there were 6–8 female offspring per exposure group per day. Experiments were repeated at least once with similar results. An * signifies a p-value ≤ 0.05 by Student’s t-test.

Unsupervised hierarchical clustering shows that although genes involved in cellular proliferation were impacted by developmental AHR activation, distinct groups of genes are affected in resting and responding CD4^+^ T cells (Fig. 3a). We observed that responding CD4^+^ T cells from offspring developmentally exposed to TCDD uniquely up-regulated a set of genes that is distinct from all other groups. The full list of DEGs involved in proliferation pathways is in Supplemental Table 2. Given that T cell clonal expansion is a fundamental aspect of mounting a strong response to infection, we further investigated whether developmental exposure changes CD4^+^ T cell proliferation using *in vivo* and *ex vivo* approaches. We developmentally exposed mice to vehicle or TCDD, and measured clonal expansion of CD4^+^ T cells specific for viral nucleoprotein (NP) peptide (311–325) after IAV challenge. Compared to offspring of control dams, the number of NP-specific CD4^+^ T cells was significantly lower 6, 9 and 12 days after IAV infection in adult offspring of TCDD-exposed dams (Fig. 3b). Nine days after infection, which is the height of the T cell response to IAV, we determined the number and percentage of proliferating CD4^+^ T cells using the marker Ki67. Consistent with fewer NP^+^CD4^+^ T cells, developmental AHR activation significantly reduced the number and percentage of proliferating CD4^+^ T cells (Fig. 3c,d).

We next determined whether this reflects impaired proliferation machinery within CD4^+^ T cells by stimulating them in culture with monoclonal antibodies that provide the two essential proliferation activation signals. Using concentrations of anti-CD3/CD28 that drive maximal proliferation, CD4^+^ T cells proliferated similarly after 4 days of stimulation, regardless of whether they were from adult offspring of dams given vehicle or TCDD (Fig. 3e). Additionally, we tested a sub-optimal concentration of anti-CD3/CD28, to determine if aberrant proliferative potential was revealed when the stimulus was not as strong. After 4 days of sub-optimal stimulation, we observed similar proliferation of CD4^+^ T cells from vehicle and TCDD offspring (Fig. 3f). CD4^+^ T cells from vehicle or TCDD exposed dams were also equally able to proliferate under maximal and sub-optimal conditions after 3 days in culture, suggesting developmental exposure does not alter the rate of proliferation (Fig. 3g,h). Taken together, these results indicate that developmental activation of the AHR diminishes CD4^+^ T cell proliferation during an *in vivo* immune challenge, but *ex vivo* mitogenic stimulation can overcome this defect. Thus, while pathways that drive T cell proliferation are affected by developmental exposure, the cell proliferation machinery within CD4^+^ T cells is operational.

### CD4^+^ T cell differentiation is impacted by developmental AHR activation

**Figure 4:**
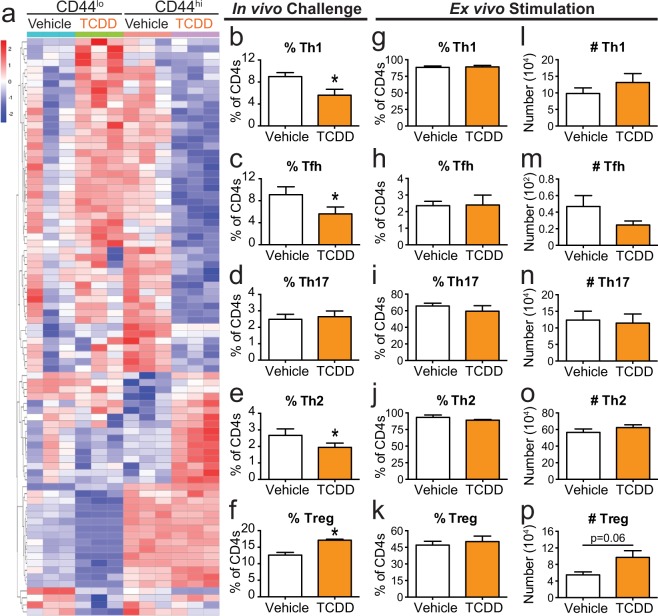
CD4^+^ T cells from mice developmentally exposed to TCDD do not have a differentiation defect in culture. (**a**) Heat map shows differentiation related DEGs. Genes are ordered using unsupervised clustering by row. See Supplemental Table 3 for gene list. (**b–f**) Offspring that were developmentally exposed to vehicle or TCDD were infected with IAV at maturity. On day 9 post-infection, the percentage of CD4^+^ T cells subsets were quantified. All subsets were gated on CD4^+^ cells, and further defined as: (**b**) Th1 Tbet^+^, (**c**) Tfh CD44^hi^CXCR5^+^PD1^+^, (**d**) Th17 RORγt^+^, (**e**) Th2 GATA3^+^, and (**f**) Treg Foxp3^+^CD25^+^. (**g**–**p**) CD4^+^ T cells from naïve offspring were cultured *ex vivo* in conditions that have been optimized to drive them toward one of these subsets. After 5 days in culture, cells were stained with fluorescent antibodies and flow cytometry was used to evaluate differentiation. (**g**–**k**) Bar graphs show the (**g**) percent of Th1, (**h**) Tfh, (**i**) Th17, (**j**) Th2, and (**k**) Tregs. (**l**–**p**) Bar graphs shows the number of (**l**) Th1, (**m**) Tfh, (**n**) Th17, (**o**) Th2, and (**p**) Tregs. Offspring from 5–7 separate dams were used per treatment group for each experiment, and experiments were repeated at least once with similar results. Values are mean ± SEM. An * signifies a p-value ≤ 0.05 by Student’s t-test.

Genes related to T cell differentiation were also altered by developmental exposure in both resting and responding CD4^+^ T cells (Fig. 4a). Interestingly, many of the genes that were up-regulated in vehicle responding CD4^+^ T were also up-regulated in resting, but not responding, CD4^+^ T cells from mice developmentally exposed to TCDD. A full list of DEGs related to differentiation can be found in Supplemental Table 3. Therefore, in addition to diminishing proliferation, the reduced number of Th1, Tfh, and Th17 cells (Fig. 1a–c) could be the result of impaired T cell differentiation. Triggering the AHR during development significantly reduced the percentage of Th1 and Tfh cells during IAV infection at adulthood (Fig. 4b,c). Compared to the two Th subtypes that predominate during acute primary IAV infection, the percentage of Th17s was not significantly different in the two groups of offspring (Fig. 4d). Often when the percentage of Th1 cells declines, there is a compensatory increase in Th2 cells. However, developmental AHR activation reduced the percentage of Th2 cells during IAV infection (Fig. 4e). There is another CD4^+^ T cell subset known as regulatory T cells (Tregs) that help maintain peripheral tolerance and promote resolution after viral infections^26^. The proper balance of immunostimulatory:immunoregulatory CD4^+^ T cell subsets is critical for a properly functioning immune system. Following developmental AHR activation, the percentage of Tregs was increased during IAV infection (Fig. 4f). Thus, consistent with prior reports, AHR activation impacts CD4^+^ T cell differentiation during IAV infection^22^.

We next sought to determine whether developmental exposure affects the ability of CD4^+^ T cells to differentiate under optimal *ex vivo* differentiation driving conditions. Rather than infecting adult offspring of treated dams, we isolated naïve CD4^+^ T cells and drove differentiation to a specific T cell subset using well established culture systems^27–33^. Developmental AHR activation did not alter the percentage or number of Th1, Tfh, Th17, or Th2 generated using *ex vivo* culture conditions (Fig. 4g–j,l–o). Also, the percentage of CD4^+^ T cells that differentiated into Tregs was not different between the two exposure groups (Fig. 4k). However, developmental AHR activation increased the number of Tregs generated *ex vivo* (Fig. 4p). This suggests that developmental AHR activation may increase the proliferative potential of inducible Tregs, which contributes to the enhanced proportion of Tregs observed *in vivo* (Fig. 4f) and^22^. More broadly, these findings indicate that when provided with optimal exogenous driving stimuli, naïve CD4^+^ T cells can overcome the impairment caused by developmental AHR activation. Overall, these results demonstrate that the distorted CD4^+^ T cell differentiation observed *in vivo* is likely due to a combination of factors that are intrinsic and extrinsic to CD4^+^ T cells.

### Developmental AHR activation impacts CD4^+^ T cell metabolism

**Figure 5:**
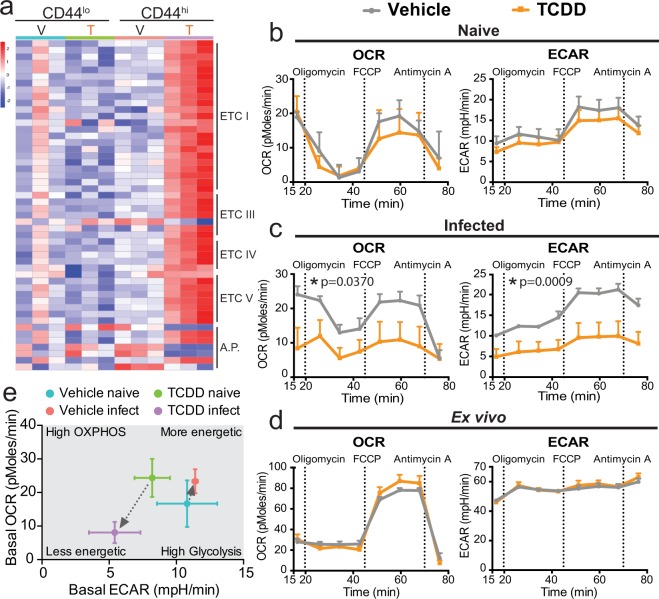
Developmental exposure to TCDD impairs mitochondrial metabolism in CD4^+^ T cells following an immune challenge later in life. (**a**) Heat map of genes that are differentially expressed following developmental AHR activation in resting and responding CD4^+^ T cells that are involved in the electron transport chain (ETC). Genes are clustered in order of ETC complex. A.P. stands for ETC accessory proteins. See Supplemental Table 4 for the list of DEGs. (**b**–**d**) A Seahorse XF^e^ analyzer was used to measure the oxygen consumption rate (OCR) and extracellular acidification rate (ECAR). Vertical dashed lines denote the addition of the indicated drug to the system. (**b**) CD4^+^ T cells were purified from naïve developmentally exposed mice and assayed immediately. (**c**) CD4^+^ T cells were purified from the MLNs 9 days after IAV infect infection. Cells were assayed immediately after purification. (**d**) CD4^+^ T cells were isolated from naïve mice, activated *ex vivo* with α-CD3/CD28, and assayed 5 days later. OCR and ECAR data are normalized to cell input (all wells contained 2 × 10^5^ CD4^+^ T cells). (**e**) Bioenergetics plot of basal OCR (y-axis) and basal ECAR (x-axis) of CD4^+^ T cells from naïve and IAV infected mice. For each experiment, 3–6 offspring from separate dams were used per treatment, and experiments were repeated at least once with similar results. All values are mean ± SEM. An * signifies a p-value ≤ 0.05 by univariate repeated-measures ANOVA.

Coordinated shifts in metabolism are critical for CD4^+^ T cell activation, differentiation, and proliferation^34,35^. Transcriptome analysis revealed genes in several different complexes that form the electron transport chain (ETC), including complex I, III, IV, and V, were differentially expressed in responding T cells from infected offspring with developmental AHR activation (Fig. 5a, Supplemental Table 4). To determine whether developmental exposure changed cellular metabolism, we measured Oxygen Consumption Rate (OCR) as an index of cellular respiration, and extracellular acidification rate (ECAR) as a surrogate index of cellular glycolysis in CD4^+^ T cells isolated from adult offspring of vehicle and TCDD exposed dams. CD4^+^ T cells from naïve vehicle and TCDD exposed offspring had similar rates of OCR and ECAR (Fig. 5b), suggesting that resting cellular metabolism was not appreciably affected. However, following *in vivo* immune challenge with IAV, the rates of both cellular respiration (OCR) and glycolysis (ECAR) were significantly lower in CD4^+^ T cells isolated from animals developmentally exposed to TCDD compared to vehicle control (Fig. 5c). Given that *ex vivo* stimulation drove a similar percentage of CD4^+^ T cells to proliferate, we next determined whether cellular metabolism was also unaffected when isolated CD4^+^ T cells were stimulated *in vitro*. These experiments revealed that *ex vivo* activated CD4^+^ T cells from vehicle and TCDD developmentally exposed offspring had similar rates of respiration and glycolysis (Fig. 5d). CD4^+^ T cells from vehicle and TCDD developmentally exposed mice had higher spare respiratory capacity when stimulated *ex vivo* versus *in vivo* stimulation with IAV infection. A majority of CD4^+^ T cells are activated after 5 days in culture, compared to ~20% activated cells during IAV infection. This difference further supports that the percentage of activated cells influences the rates of oxidative phosphorylation. In response to an immune challenge CD4^+^ T cells typically up regulate these metabolic pathways to sustain high rates of proliferation. Yet, CD4^+^ T cells from adult mice that were developmentally exposed to TCDD down regulated both cellular respiration and glycolysis (Fig. 5e). These differences in cellular bioenergetics suggest that one mechanism by which developmental AHR activation reduces CD4^+^ T function is impaired cellular metabolism.

**Figure 6:**
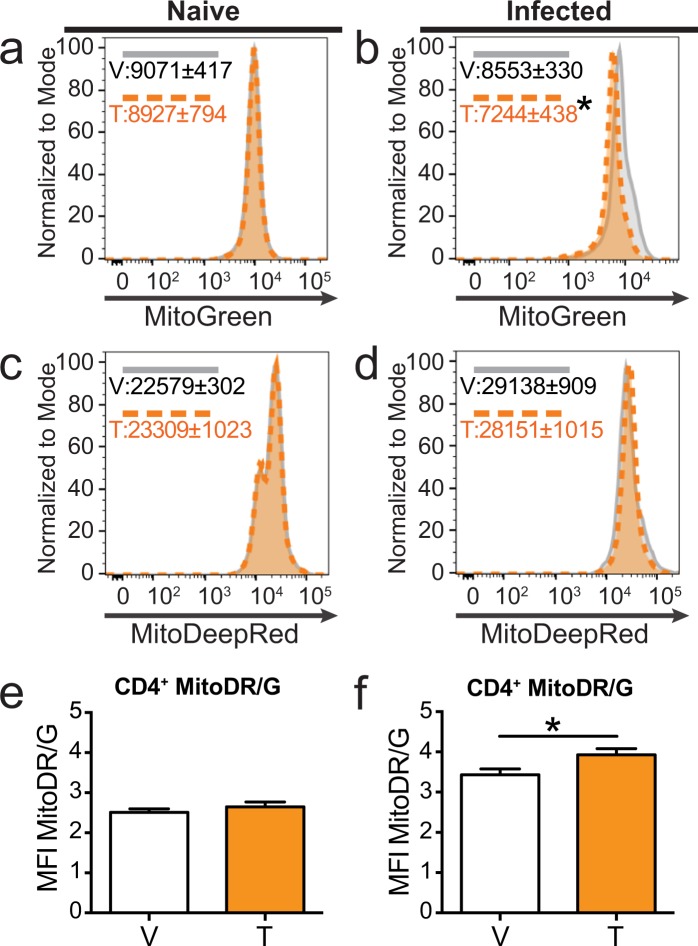
Mitochondrial mass is reduced in CD4^+^ T cells following developmental AHR activation and IAV infection later in life. (**a**,**c**,**e**) Peripheral lymph nodes from naïve mice or (*B*,*D*,*F*) MLNs from mice infected with IAV for 9 days were harvested. (**a**–**d**) Representative histograms of (*A*,*B*) Mitotracker Green and (**c**,**d**) MitoTracker Deep Red depict expression of each dye in CD4^+^ T cells from vehicle (grey solid line) and TCDD (orange dashed line) developmentally exposed mice. Numbers on the histograms depict the mean ± SEM of the mean fluorescence intensity (MFI). (**e**,**f**) Bar graphs show the ratio of MitoTracker Deep Red to MitoTracker Green in CD4^+^ T cells from (**e**) naïve or (**f**) infected mice. Data are from one experiment that is representative of two independent experiments. All values are mean ± SEM and represent 6–7 offspring per treatment group. All offspring within a group are from a separate dam. Statistics used are two tailed unpaired t-tests. An * signifies a p-value ≤ 0.05.

Shifts in mitochondrial dynamics can impact the rates of cellular metabolism. Genes involved in regulating mitochondrial fission, fusion, and mitophagy were altered by developmental AHR activation, particularly in activated CD4^+^ T cells (Table 1). To take a closer look at the mitochondria, we used MitoTracker Green, which labels mitochondria independent of membrane potential and provides a relative indicator of mitochondrial mass^36^. We also used MitoTracker DeepRed, which is dependent on mitochondrial membrane potential, providing a probe for actively respiring mitochondria^37^. CD4^+^ T cells from naïve offspring developmentally exposed to vehicle or TCDD had the same mitochondrial mass (Fig. 6a), but following IAV infection developmental AHR activation significantly reduced mitochondrial mass (Fig. 6b). In naïve and infected mice, the levels of actively respiring mitochondria were similar in CD4^+^ T cells from vehicle and TCDD developmentally exposed mice (Fig. 6c,d). The ratio of actively respiring mitochondria to mitochondrial mass was increased in CD4^+^ T cells from mice developmentally exposed to TCDD after, but not before, immune challenge (Fig. 6e,f). These results suggest developmental AHR activation increased mitochondrial polarization. Collectively, these results indicate that one mechanism by which developmental AHR activation alters CD4^+^ T cells responses later in life is by altering cellular metabolism and mitochondrial dynamics.

**Table 1:** Developmental AHR activation affects genes that regulate mitochondrial fission, fusion, and mitophagy.

Gene	Function	Log_2_ Fold Change	Adjusted p-value
*Fis1*	Promotes fission	1.07	0.0019
*Mff*	Promotes fission	1.21	0.0075
*Cycs*	Released following fission	1.02	0.0017
*Mfn2*	Promotes fusion	−1.23	0.0124
*Atg2b*	Regulates autophagy/mitophagy	−1.09	0.0277
*Atg2a*	Regulates autophagy/mitophagy	−1.16	0.0100
*Atg7*	Regulates autophagy/mitophagy	−1.59	0.0083

DEGs in responding CD4^+^ T cells and that gene’s function related to mitochondrial fission, fusion, or authophagy/mitophagy are shown. The log_2_ fold change and adjusted p-value for each gene is listed. An increase in fold change indicates developmental AHR activation increases expression of that gene in CD4^+^ T cells.

## Dicussion

Developmental periods are sensitive to environmental cues. Early life exposures profoundly shape health trajectories and can contribute to disease later in life. This concept, often referred to as the “developmental basis of adult disease,” has been linked to the risk of developing cancer, infertility, diabetes, cardiovascular disease, and obesity^38–40^. Although it has received less attention, early life environmental factors also influence the immune system, with emerging evidence that environmental exposures reduce vaccine responses and increase the severity and incidence of infectious diseases^5–12^. Yet, the cellular mechanisms by which most developmental exposures trigger persistent changes in immune function are uncertain. One means by which immune cells sense environmental cues is via the AHR. Early life AHR activation, via maternal exposure to the prototype AHR agonist TCDD, leads to alterations in CD4^+^ T cell responses to a common respiratory pathogen^22^. Since children exposed to AHR ligands during early life have more respiratory tract infections and increased wheezing^5,6,8,11,12^, we focused on CD4^+^ T cells because this cell type contributes to the immune responses that underlie fighting respiratory infections and developing chronic pulmonary inflammatory diseases. Additionally, CD4^+^ T cell dependent immune responses are influenced by other early life environmental insults, such as cigarette smoke, trichloroethylene, and arsenic exposures, suggesting that they may be sentinels for early life exposures impacting immune function later in life^22,24,41–43^. However, the mechanisms by which early life exposures exert lasting changes remain unclear. In the present study, we demonstrated that developmental exposure to an AHR ligand targeted multiple cellular pathways that are critical for CD4^+^ T cell function, including, but not limited to, proliferation, differentiation, and metabolism.

One of the interesting findings from the current study was that T cell stimulation *in vivo* and *ex vivo* did not yield similar outcomes. All *ex vivo* experiments were performed using maximal stimulating conditions to test the potential ability of CD4^+^ T cells from developmentally exposed mice to respond. It is possible that earlier points in time or using suboptimal stimuli may yield slightly different results. Yet, other studies have reported analogous disconnects between *ex vivo* and *in vivo* systems in the context of direct exposure to AHR ligands in adult animals, suggesting a more nuanced explanation^44–49^. For example, animals directly exposed to TCDD have reduced serum cytokine levels when they were challenged *in vivo* with an anti-CD3 injection^49^. However, when splenocytes are isolated from TCDD exposed animals and stimulated in culture with anti-CD3, cytokine levels were not changed^49^. Cells of the immune system do not act in isolation, and this is especially true for CD4^+^ T cells, whose responses are dependent on interactions with other cell types and the microenvironment. Among the cell types that play important roles during IAV infection are T cells, B cells, dendritic cells, and non-immune cells, which all express the AHR^50–52^. Previous work has shown that, in addition to affecting CD4^+^ T cells, developmental AHR activation influences CD8^+^ T cells and dendritic cells^22–24,53,54^. Moreover, other studies indicate that developmental exposure likely influences CD4^+^ T cell responses by a combination of cell autonomous and external factors^22,24^. The present study reveals that CD4^+^ T cell proliferation, differentiation, and metabolism are all affected by an *in vivo* challenge. However, these defects are overcome by stimulation *ex vivo*. This suggests that developmental activation of the AHR diminishes CD4^+^ T cell responses *in vivo* by impacting steps critical for CD4^+^ T cell activation. This potentially occurs due to the impaired ability of T cells to respond to environmental cytokines, interact with APCs via co-stimulatory molecules, and/or recognize cognate peptide: MHC II complexes. This hypothesis is further supported by adoptive transfer studies showing that developmentally exposed CD4^+^ T cells transferred into unexposed host exhibit impaired responses during IAV challenge^22^. Together, these results highlight that in order to fully grasp how environmental exposures influence different immune cell populations, we need to continue assessing their function within an intact *in vivo* system.

**Figure 7:**
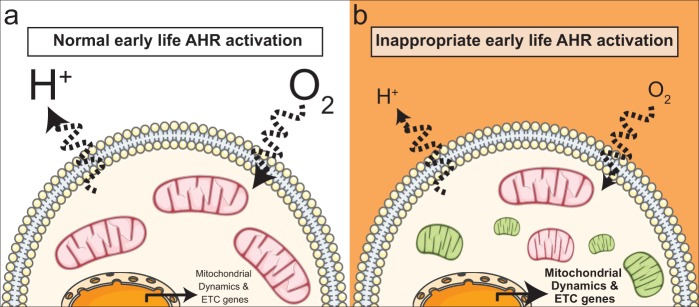
Developmental AHR activation impacts mitochondrial metabolism and dynamics. (**a**) The cartoon depicts aspects of CD4^+^ T cell responses during IAV infection in the absence of maternal exposure to exogenous, environmental AHR ligands. (**b**) Inappropriate AHR activation during development impacts cellular metabolism. Specifically, early life AHR activation decreases OXPHOS (O_2_) and glycolysis (H^+^) rates, increases transcription of electron transport chain (ETC) components, increases mitochondrial fission, and dampens fusion. This leads to the accumulation of smaller mitochondria. Pink mitochondria are healthy, and green mitochondria are damaged or dysfunctional. This figure was created using Servier Medical Art (https://smart.servier.com. License, which are licensed under the Creative Commons Attribution 3.0 Unported License agreement (https://creativecommons.org/licenses/by/3.0/).

Among the changes in T cells, a key novel finding of the present study is that cellular metabolism is influenced by early life AHR activation. Specifically, early life AHR activation reduces the rates of cellular respiration and glycolysis in CD4^+^ T cells (Fig. 7). All of the metabolic assays performed in this study used total CD4^+^ T cells. An interesting further area of investigation is whether developmental AHR activation influences cellular metabolism in an activation state or subset specific manner. CD4^+^ T cells undergo critical shifts in cellular metabolism when they become activated, differentiate into conventional helper cells, or differentiate into regulatory T cells^35,55–58^. Thus, understanding whether developmental AHR activation targets the key metabolic features required for activation or differentiation will shed light on the mechanisms driving impaired immune responses following developmental exposure.

Another intriguing finding from this study is that developmental AHR activation may influence mitochondrial dynamics. There was also an increase in mitochondrial fission gene expression, with a concurrent decrease in expression of genes related to mitochondrial fusion. Fission creates new mitochondria during cell division, and eliminates damaged mitochondria^59,60^. On the other hand, fusion combines two or more mitochondria to reduce cellular stress by diluting partially damaged mitochondria with properly functioning mitochondria^59,60^. Fusion of partially damaged mitochondria, or recycling via mitophagy, is largely controlled by mitochondrial membrane potential^59,60^. Our data suggest that CD4^+^ T cells from offspring that were developmentally exposed to TCDD have increased membrane potential, which can reduce mitophagy events^61^. Consistent with this idea, transcriptome analysis revealed that genes critical for mitophagy are reduced in responding CD4^+^ T cells from developmentally exposed mice. Combined, this implies that developmental AHR activation might increase fission to eliminate damaged mitochondria; however, damaged mitochondria accumulate because mitophagy and fusion are reduced (Fig. 7). Further investigation is needed to better understand how these changes affect mitochondrial functions, but our observations imply that developmental exposure may impair cellular metabolism through altered quality control mechanisms^62–64^.

We also observed that genes involved in 4 out of the 5 ETC complexes are increased in responding CD4^+^ T cells after developmental AHR activation (Fig. 7). At first glance, this may seem incongruous with the decrease in oxidative phosphorylation that was observed. However, others have observed an increase in ETC genes with a decrease in cellular metabolism^65–67^. Increased expression of ETC component genes correlated with signals that reduce autophagy and apoptosis^65,67^. Thus, increased transcription of ETC components could aid in preventing cell death. Additionally, an increase in ETC genes has been proposed as a compensatory mechanism to boost mitochondria biogenesis in response to damaged, condensed, and collapsed mitochondria^66^. We observed a reduction in mitochondrial mass; however, the dye used specifically labels the matrix^36^. If developmental exposure caused condensation or collapse of mitochondrial cristae, the morphology of the mitochondria would be severely altered, which would impact labeling in the matrix^36,66^. Additionally, other factors can influence MitoTracker dye labeling, including mitochondrial number, mitochondria size, and ROS production. Despite these limitations, it is likely that multiple of these mechanisms explain why we observed an increase in ETC genes and a decrease in cellular metabolism. Further, this suggests that developmental AHR activation may alter regulation of mitochondrial biogenesis. Together, these results suggest that developmental AHR activation might impact mitochondrial dynamics, but further study is needed to understand these changes. Shifts in mitochondrial dynamics are critical to imparting cell fate decisions in T cells; therefore, any alterations would have dramatic effects on their energy production and responsive capacity^68^.

Another intriguing observation of this study is that diminished CD4^+^ T cell functions, due to early life AHR activation are only revealed after the immune system is triggered to respond. Early life AHR activation also changes the response of CD4^+^ T cell during non-infectious immune challenges^24^. Additionally, early life exposure in humans durably changes T cell dependent immune responses, such as antibody responses to vaccines^7,9^. Yet, many human studies tend to evaluate the effects of early life exposures exclusively in infants and young children. Few studies have followed these infants into adolescence or adulthood; however, one recent study observed an association between early life exposure to higher levels of perfluorinated alkylate substances (PFASs) with reduced vaccine-specific antibody levels at 13 years of age^69^. Together, this calls for longer-term epidemiology studies to better understand the full ramifications of early life exposures on the function of the immune system, because some dormant alterations may not be revealed until immune stressors are triggered later in life.

Although changes to cellular functions are only revealed after an immune challenge, our results indicate one way that developmental AHR activation programs impaired responses is by causing lasting changes to gene expression patterns. Yet, we still do not understand which mechanisms are driving these long-term effects. Compared to vehicle controls, mice developmentally exposed to TCDD have significantly increased expression of AHR target genes shortly after birth. However, at maturity these genes are no longer elevated, indicating that there is not sustained AHR activation as mice mature to adulthood^54^. This is consistent with the half-life of TCDD in mice, which is approximately 7 days^70,71^, supporting that developmentally mature offspring have no residual TCDD. Thus, environmental exposure to AHR ligands programs cellular responses early during the development of the immune system. Since these changes are long lasting and programmed during early development, it implicates altered epigenetic regulation as a potential mechanism. One epigenetic mechanism that can be influenced by environmental cues to alter cellular function, and is important for the development of the immune system is DNA methylation^72,73^. Although the exact mechanisms that drive impaired CD4^+^ and CD8^+^ T cell responses after developmental AHR activation are likely not identical, developmental activation of the AHR durably shifts DNA methylation patterns in CD8^+^ T cells in mice^54^. Additionally, these DNA methylation changes correlate with altered gene expression and CD8^+^ T cell function^54^. In other studies, exposure to trichloroethylene alters cellular function and DNA methylation in CD4^+^ T cells^74,75^. Thus, the impaired responsive capacity of CD4^+^ T cells that results from developmental AHR activation may also reflect changes in DNA methylation.

Given that CD4^+^ T cells are central to appropriate immune responses to many pathogens and to the effectiveness of vaccines, these findings provide a deeper understanding of how early life exposures to chemicals that bind the AHR durably shape immunity later in life. For instance, CD4^+^ T cells play a central role in generating and maintaining immunological memory. Therefore, their altered function is likely a driver of impaired ability to cope with primary infection, and a reduced ability to fight repeated infections and lower vaccine responses. This prediction is borne out of several human studies that reported an association between increased incidence of infections and decreased vaccine responses with early life exposure to environmental AHR ligands^5,7,69,76–79^. Furthermore, these results have implications beyond infectious disease, because CD4^+^ T cells are involved in antitumor immunity, and dysregulated CD4^+^ T cell function contributes to autoimmune and allergic diseases. The incidences of these diseases are on the rise in industrialized developed countries. Given that industrial by-products are a major source of environmental AHR ligands, and that developmental AHR activation influences the responsive capacity of CD4^+^ T cells, altered CD4^+^ T cell function could contribute to the rise of these diseases. By better understanding which cell types and cellular functions are targeted by early life environmental exposures, we can devise better-targeted vaccine and treatment strategies for people at high risk of exposure.

## Materials and Methods

### Animals models

C57BL/6 (*Ahr*^*b/b*^) mice (age 5–6 weeks) were obtained from Jackson Laboratory (Bar Harbor, ME). Nulliparous C57BL/6 females were housed with C57BL/6 males, and checked daily for the presence of a vaginal plug, which was designated as day 0 of gestation. 2,3,7,8-Tetrachlorodibenzo-*p*-dioxin (TCDD; ≥99% purity; Cambridge Isotope Laboratories, Woburn, MA) was dissolved in anisole and diluted in peanut oil. The vehicle control consisted of peanut oil containing an equivalent concentration of anisole (0.01%). Impregnated female mice were treated with 1 µg TCDD /kg body weight (BW) or the peanut oil vehicle control (vehicle) by oral gavage on days 0, 7, and 14 of gestation, and 2 days after parturition. Offspring from treated dams were weaned 3 weeks post birth. Mice were housed in microisolator cages in a specific-pathogen free facility at the University of Rochester Medical Center, and were provided food and water ad libitum. Supplemental Table 1 shows some of the key CD4^+^ T cell responses during IAV infection in male and female developmentally exposed mice. This is consistent with prior reports that developmental exposure to TCDD triggers similar changes to the immune response to IAV in male and female offspring^23^. All experiments presented herein were performed using female developmentally exposed offspring. All animal treatment was conducted with prior approval from the Institutional Animal Care and Use Committee of the University of Rochester, and studies were performed in accordance with all guidelines and regulations.

### Influenza virus infection

Influenza A virus (HKx31) was prepared and titered as previously described^80^. Adult offspring (6–10 weeks of age) were anesthetized with an intraperitoneal (i.p.) injection of avertin (2,2,2-tribromoethanol; Sigma Aldrich, St. Louis, MO). Mice were infected intranasally (i.n.) with 120 hemagglutination units (HAU) of influenza A virus (IAV), which is a sublethal inoculum. All work with infectious agents was conducted with prior approval of the Institutional Biosafety Committee of the University of Rochester, following guidelines of the NIH/CDC.

### CD4^+^ T cell purification

Single cell suspensions were harvested from peripheral lymph nodes (cervical, axillary, brachial, inguinal, and sacral) of naïve adult, age matched, female developmentally exposed mice. CD4^+^ T cells were purified to >90% purity using the MojoSort Mouse CD4^+^ T cell negative selection isolation kit protocol (BioLegend, San Diego, CA). Additionally, for RNA-seq single cell suspensions from MLNs of day 9 post IAV infected developmentally exposed offspring were stained with fluorescently conjugated antibodies to CD3, CD4, and CD44. Stained cells were FACS purified into CD4^+^CD3^+^CD44^lo^ (resting) or CD44^hi^ (responding) cells. Thus CD44 expression level was used to distinguish resting and responding CD4 T cells^81^.

### RNA-sequencing

For each RNA-seq replicate, CD4^+^ T cells from 8–9 female offspring from 3–4 unique dams were pooled to ensure ample material was isolated to perform RNA-seq. Offspring from the same dam used in only a single pool of cells. In total, this required the use of offspring from 22 dams. This created 6 unique pools of cells prior to FACs purification. RNA was isolated from purified T cells using the RNeasy Mini kit (Qiagen, Valencia, CA). Total RNA concentration was determined with the NanopDrop 1000 spectrophotometer (NanoDrop, Wilmington, DE) and RNA quality assessed with the Agilent Bioanalyzer 2100 (Agilent, Santa Clara, CA). RNA (1 ng) was pre-amplified with the SMARTer Ultra Low RNA Kit for Illumina Sequencing (Clontech, Mountain View, CA) following the manufacturer’s recommendations. The quantity and quality of the subsequent cDNA was determined using the Qubit Flourometer (Life Technnologies, Carlsbad, CA) and the Agilent Bioanalyzer 2100 (Agilent, Santa Clara, CA). cDNA (1 ng) was used to generate Illumina compatible sequencing libraries with the NexteraXT library preparation kit (Illumina, San Diego, CA). The amplified libraries were hybridized to the Illumina single end flow cell, and amplified using the cBot (Illumina, San Diego, CA) at a concentration of 10 pM per lane. Single end reads of 100nt were generated for each sample using Illumina’s HiSeq2500v4. All sequence data are available from the Gene Expression Omnibus (http://www.ncbi.nlm.nih.gov/geo/query/acc.cgi?acc=GSE1234229) under accession number GSE134229).

### RNA-sequencing computational analysis

Raw reads generated from the Illumina HiSeq. 2500 sequencer were demultiplexed using configurebcl2fastq.pl version 1.8.4. Quality filtering and adapter removal are performed using Trimmomatic version 0.32 with the following parameters: “SLIDINGWINDOW:4:20 TRAILING:13 LEADING:13 ILLUMINACLIP:adapters.fasta:2:30:10 MINLEN:15”^82^. Processed/cleaned reads were then mapped to the mouse reference genome (GRCm38.p4 + Gencode-M6 Annotation) using STAR_2.4.2a with the following parameters: “–twopassMode Basic–runMode alignReads–outSAMtype BAM SortedByCoordinate–outSAMstrandField intronMotif–outFilterIntronMotifs RemoveNoncanonical–outReadsUnmapped Fastx”^83^. Gene-level read quantification was derived using htseq-count 0.6.1 with a GTF annotation file (Gencode M6) and the following parameters: “-q -f bam -s no -r pos -i gene_name”^84^. Differential expression analysis was performed using DESeq2–1.12.4 with an adjusted p-value threshold of 0.05 within R version 3.3.0^85^. Heatmaps were created within R using the pheatmap package given rLog transformed expression values^86^. Genes were considered differentially expressed (DEGs) if they had an adjusted p-value < 0.05 and absolute fold change of >2. Pathway analysis was performed on DEGs using Ingenuity Pathways Analysis (IPA; Ingenuity Systems, http://www.ingenuity.com).

### *Ex vivo* proliferation assay

Purified CD4^+^ T cells from naïve animals were stained with 2 μM CFSE (Molecular Probes, Inc. Eugene, OR). Cells were stimulated with anti-CD3 (clone: 145-2C11, eBiosceince, San Diego, CA) and anti-CD28 (clone: 37.51, eBiosceince, San Diego, CA) for 3–4 days in culture with RPMI media containing 10% FBS, 1X Glutamax, 50 μM 2-mercaptoethanol, and 1X pen/strep. 5 μg/mL or 2.5 μg/mL of anti-CD3/CD28 were used for maximal or sub-optimal stimulation; respectively. Cells were harvested, stained with fluorescent antibodies, and analyzed via flow cytometry.

### *Ex vivo* differentiation assay

Purified CD4^+^ T cells from naïve mice were cultured in the following subset driving conditions: Th1: 5 μg/mL anti-IL4 (clone 11B11) + 5 ng/mL IL-12, Th2: 5 μg/mL anti-IFNγ (clone XGB1.2) + 4 ng/mL IL-4, Th17: 5 μg/mL anti-IL4 + 5 μg/mL anti-IFNγ + 20 ng/mL IL6 + 1 ng/mL TGFβ + 10 ng/mL IL23, Tfh: 5 μg/mL anti-IL4 + 5 μg/mL anti-IFNγ + 10 μg/mL anti-TGFβ (clone 1D11) + 100 ng/mL IL6 + 50 ng/mL IL21, and Treg: 5 μg/mL anti-IL4 + 5 μg/mL anti-IFNγ + 5 ng/mL TGFβ + 10 ng/mL IL2. Th1, Th2, Th17, and Treg cultures were in RPMI media containing 10% FBS, 1X Glutamax, 50 μM 2-mercaptoethanol, and 1X pen/strep. The base for Tfh driving media was DMEM, containing 10% FBS, 1X Glutamax, 50 μM 2-mercaptoethanol, and 1X pen/strep. After 5 days in culture, cells were harvested and differentiation was evaluated using flow cytometry.

### Cellular metabolism

A Seahorse XF^e^96 metabolic extracellular flux analyzer (Agilent Technologies, Santa Clara, CA) was used to measure oxygen consumption rate (OCR) and extracellular acidification rate (ECAR). CD4^+^ T cells were purified from peripheral LNs of naïve mice or MLNs of IAV infected developmentally exposed offspring. CD4^+^ T cells (2 × 10^5^ per well) were seeded in XF96 cell culture microplates (Agilent Technologies, Santa Clara, CA). All data are normalized to cell input. Prior to adding cells, the microplates were coated with 100 μg/mL poly-D-lysine hydrobromide (Corning, Two Oak Park, Bedford, MA). Cells were suspended in Seahorse XF media (Agilent Technologies, Santa Clara, CA) containing 10 mM glucose, 1 mM pyruvate, and 2 mM Glutamax. To measure the metabolic prolife of the cells, 1 μM oligomycin A, 1.5 μM carbonyl cyanide-4-(trifluoromethoxy)phenylhydrazone (FCCP), and 1 μM antimycin A (all from Sigma Aldrich; Saint Louis, MO) were sequentially injected into the wells. Oligomycin blocks ATP synthase, FCCP uncouples the proton gradient, and Antimycin A blocks ETC complex III. OCR and ECAR were measured every 7 minutes for 90 minutes.

### Flow cytometry

Nonspecific staining was blocked using anti-mouse CD16/32 mAb. The following fluorescently conjugated antibodies against cell-surface antigens were used: CD3e (clone145-211), CD4 (clone RM4-5), CD25 (clone PC61.5), CD44 (clone IM7), CD62L (clone MEL-14), CXCR5 (Biotin, with PE-conjugated streptavidin, clone 2G8), and PD-1 (clone J43). Cells were incubated with major histocompatibility class II tetramers containing an immunodominant peptide epitope of HK × 31 (nucleoprotein, I-A^b^/NP_311–325_; NIH Tetramer Core Facility, http://tetramer.yerkes.emory.edu/) to identify virus specific CD4^+^ T cells. For intracellular staining, cells were fixed and permeabilized with Foxp3 Staining Kit (eBiosience, San Diego, CA), and incubated with fluorochrome-conjugated antibodies against Ki67 (clone B56), TBet (clone 4IBO), GATA3 (clone L50-823), RORγt (clone Q31-378), Foxp3 (clone FJK-16S), as described previously^22^. The following subset specific markers were used to identify Th1 (Tbet^+^), Th2 (GATA3^+^), Th17 (RORγt^+^), Tfh, (CD44^hi^CXCR5^+^PD1+) and Treg (Foxp3^+^CD25^+^) CD4^+^ T cells. To stain mitochondria we used 100 nM MitoTracker Green and 100 nM MitoTracker Deep Red (both from Molecular Probes, Inc. Eugene, OR). MitoTracker Green labels all mitochondria independent of membrane potential, and MitoTracker Deep Red labeling is dependent on membrane potential. All antibodies were purchased from eBioscience (San Diego, CA) or BD Biosciences (San Jose, CA). Fluorescence minus one (FMO) controls were used to determine non-specific fluorescence and define gating parameters. Data were collected using an LSRII flow cytometer (BD Biosciences, San Jose, CA), and analyzed using the FlowJo software program (TreeStar, Ashland, OR).

### Statistical analysis

For all experiments, the dam is defined as the statistical unit. Offspring used in each treatment group and at each time point relative to infection were from a different treated dam. Except for RNASeq data, all data were analyzed using JMP software (SAS Institute Inc., Cary, NC). Differences between exposure groups were evaluated using the statistical test indicated in each figure legend. Differences were considered significant if p ≤ 0.05. All data are presented as mean ± SEM.

## Supplementary information

Supplementary Data Tables

## Data Availability

All sequence data are available from the Gene Expression Omnibus (http://www.ncbi.nlm.nih.gov/geo/query/acc.cgi?acc=GSE1234229). The other datasets generated and analyzed during the current study are available from the corresponding author upon reasonable request.
