# Supplementary material for: Early life exposures shape the CD4+ T cell transcriptome, influencing proliferation, differentiation, and mitochondrial dynamics later in life
Source: Sci Rep. 2019 Aug 7;9:11489. doi: 10.1038/s41598-019-47866-2 (PMC6686001; doi:10.1038/s41598-019-47866-2)
Supplement: Supplementary file 1 — Supplementary Data Tables [file 41598_2019_47866_MOESM1_ESM.pdf]

SUPPLEMENTARY MATERIALS

TITLE PAGE

Title: Early life exposures shape the CD4$^+$ T cell transcriptome, influencing proliferation, differentiation, and mitochondrial dynamics later in life

Authors: Catherine G. Burke[1], Jason R. Myers[2], Lisbeth A. Boule[1], Christina M. Post[3], Paul S. Brookes[4], and *B. Paige Lawrence[1,3]

Departments of [1]Microbiology & Immunology, [2]Genomics Research Center, [3]Environmental Medicine, [4]Anesthesiology, University of Rochester School of Medicine & Dentistry, Rochester, NY 14624

***Corresponding Author:**
B. Paige Lawrence, PhD
University of Rochester School of Medicine and Dentistry
601 Elmwood Ave, Box EHSC
Rochester, NY 14642
(585) 276-3873
Paige_Lawrence@URMC.Rochester.edu

Supplemental Table 1: CD4⁺ T cell responses in the MLN of female and male developmentally exposed mice infected with IAV at adulthood.

	Female		Male	
**$CD4^+CD3^+$ T cells**	**Mean ± SEM**	**p-value**	**Mean ± SEM**	**p-value**
Number (10^5)	V: 8.83 ± 0.7<br>T: 6.37 ± 1.1	0.04*	V: 7.98 ± 1.7<br>T: 4.59 ± 0.7	0.05*
Percent<br>(of MLN cells)	V: 16.4 ± 1.5<br>T: 19.7 ± 1.2	0.05*	V: 14.6 ± 1.0<br>T: 15.1 ± 1.1	0.37
**Virus specific NP^+ $CD4^+$ T cells**	**Mean ± SEM**	**p-value**	**Mean ± SEM**	**p-value**
Number (10^4)	V: 0.69 ± 0.1<br>T: 0.47 ± 0.1	0.07	V: 0.67 ± 0.1<br>T: 0.41 ± 0.1	0.08
Percent<br>(of $CD4^+$ cells)	V: 0.84 ± 0.1<br>T:0.76 ± 0.1	0.30	V: 0.85 ± 0.1<br>T: 0.87 ± 0.1	0.46
**Th1 ($Tbet^+CD4^+$) cells**	**Mean ± SEM**	**p-value**	**Mean ± SEM**	**p-value**
Number (10^4)	V: 8.03 ± 1.1<br>T: 3.96 ± 0.9	0.01*	V: 5.03 ± 1.2<br>T: 2.52 ± 0.6	0.05*
Percent<br>(of $CD4^+$ cells)	V: 8.99 ± 0.7<br>T: 5.60 ± 1.1	0.01*	V: 5.59 ± 0.6<br>T: 4.54 ± 0.5	0.09

Supplemental Table 1: Developmental AHR activation impairs $CD4^+$ T cell responses during IAV infection in male and female developmentally exposed offspring. Mice were developmentally exposed to vehicle or TCDD. At adulthood, offspring were infected with IAV. MLNs from female and male offspring were collected on day 9 post infection. MLNs were processed to single cell suspensions, and cells were stained with fluorescent antibodies. The number and percentage of $CD4^+$, virus specific (NP^+) $CD4^+$ T cells, and Th1 ($Tbet^+CD4^+$) T cells were enumerated using flow cytometry. Values shown are mean ± SEM and 6-7 offspring per sex per treatment group were used. Statistics used are one tailed unpaired t-tests. An * signifies a p-value ≤ 0.05.

Supplemental Table 2: Differentially expressed genes in resting and responding CD4+ T cells that are involved in proliferation pathways

	TCDD resting vs. Vehicle resting		TCDD responding vs. Vehicle responding	
**Proliferation Genes**	**Log_2 Fold Change**	**Adjusted p-value**	**Log_2 Fold Change**	**Adjusted p-value**
Pole	2.262	0.005	0.201	0.864
Cyfip1	1.637	0.001	-0.571	0.299
Cdc25b	0.344	0.686	-1.131	0.007
Il2rb	0.727	0.229	-1.659	0.000
Pla2g6	0.481	0.736	-1.379	0.053
Hdac10	0.287	0.830	-1.192	0.038
Inpp5k	0.104	0.916	-1.079	0.002
Tdp1	-0.175	0.905	-1.604	0.003
Eif4g1	0.219	0.782	-1.124	0.001
Tln1	0.169	0.847	-1.017	0.005
Trp53	-0.036	0.975	-1.764	0.000
Ets1	0.358	0.533	-1.317	0.000
Pld3	0.139	0.856	-1.103	0.000
Kat5	0.116	0.953	-1.713	0.008
Csnk1d	1.019	0.064	-1.309	0.002
Tyk2	0.942	0.189	-1.046	0.044
Mta2	0.532	0.480	-1.112	0.012
Tnfrsf1a	0.175	0.899	-1.571	0.002
Hdac8	1.555	0.082	-1.418	0.043
Pikfyve	1.267	0.001	-0.180	0.728
Akt1	1.011	0.010	-0.617	0.082
Smpd4	1.112	0.050	-0.581	0.267
Rad9a	0.375	0.706	-1.719	0.000
Itpr3	0.307	0.728	-1.188	0.005
Mef2d	0.581	0.351	-1.198	0.002
Il2rg	0.408	0.354	-1.191	0.000
Smurf1	0.186	0.847	-1.156	0.003
Tsc1	0.842	0.110	-1.025	0.009
Mknk1	0.854	0.166	-1.265	0.003
Rassf1	0.724	0.404	-1.640	0.001
Brat1	0.080	0.966	-1.217	0.052
Hdac4	0.665	0.310	-1.005	0.020
Inpp5b	0.568	0.355	-1.176	0.002
Shc1	0.520	0.512	-1.080	0.019

Fnbp1	0.508	0.436	-1.122	0.003
Rps6kb2	1.141	0.097	-1.195	0.023
Smad1	-0.336	0.795	1.383	0.018
Smad7	-0.551	0.534	1.107	0.030
Rras2	-0.316	0.826	1.378	0.026
Rpl5	-0.299	0.646	1.248	0.000
Rps19	-0.449	0.465	1.385	0.000
Rps4x	-0.548	0.258	1.222	0.000
Rps15	-0.663	0.197	1.519	0.000
Rps8	-0.453	0.406	1.411	0.000
Rps28	-0.104	0.873	1.184	0.000
Rps29	-0.306	0.569	1.381	0.000
Rps25	-0.253	0.718	1.360	0.000
Rps21	-0.243	0.720	1.636	0.000
Rps24	-0.135	0.895	1.571	0.000
Rap1b	0.096	0.909	1.113	0.000
Terf2ip	0.310	0.744	1.222	0.006
Cycs	-0.486	0.366	1.020	0.002
Rpa3	-0.190	0.877	1.605	0.001
Rps12	-0.081	0.935	1.583	0.000
Rps27l	-0.393	0.612	1.399	0.000
Anapc13	-0.455	0.533	1.262	0.001
Ppp1r14b	-0.150	0.906	1.460	0.002
Dbf4	-0.663	0.464	1.151	0.035
Rheb	-0.408	0.678	1.272	0.009
Ppp2cb	-0.213	0.877	1.184	0.033
Eif3f	-0.636	0.222	1.481	0.000
Eif3k	-0.805	0.011	1.056	0.000
Rps27a	-0.378	0.575	1.479	0.000
Rps15a	-0.446	0.428	1.585	0.000
Rps26	-0.476	0.355	1.400	0.000
Rps13	-0.258	0.666	1.045	0.000
Rps17	-0.401	0.483	1.291	0.000
Rps2	-0.466	0.466	1.472	0.000
Rps18	-0.691	0.161	1.467	0.000
Rps23	-0.538	0.329	1.528	0.000
Rps14	-0.642	0.254	1.495	0.000
Rps16	-0.566	0.245	1.308	0.000
Rps20	-0.643	0.197	1.499	0.000
Rps7	-0.764	0.131	1.521	0.000
Rpl11	-0.647	0.153	1.388	0.000

Rps5	-0.581	0.180	1.168	0.000
Rps6	-0.612	0.161	1.136	0.000
Rps9	-0.621	0.105	1.099	0.000
Fau	-0.809	0.058	1.330	0.000
Rpsa	-0.565	0.209	1.036	0.000
Cks2	-0.055	0.977	1.226	0.044
Dna2	-1.951	0.031	0.066	NA
Actg2	-1.877	0.017	0.036	0.974
Ccnb1	-2.965	0.000	0.074	0.933
Lig1	-1.179	0.002	0.354	0.377
Bmp7	-1.930	0.030	-0.116	0.927
Bhlhe40	-2.836	0.000	-0.984	0.157
Cybb	-3.760	0.000	-0.679	0.302
Aurka	-1.748	0.050	0.096	0.939
Plcb4	-2.872	0.000	-0.471	0.525
Plk1	-2.085	0.009	-0.920	NA
Bad	-1.173	0.044	0.421	0.471
Mef2c	-2.998	0.000	-0.143	0.898
Rhoc	-2.029	0.027	0.560	NA
Hdac2	-1.246	0.038	0.668	0.225
Map2k6	-2.157	0.009	-1.011	NA

Supplemental Table 2: Developmental AHR activation changes expression of genes related to proliferation. The $\log_2$ fold change and adjusted p-value for each gene are listed, comparing gene expression in resting and responding T cells from vehicle and TCDD exposure groups. An increase in fold change indicates developmental AHR activation increases expression of that gene in $CD4^+$ T cells. Genes were ordered using unsupervised clustering by row, and are listed from top to bottom in the order that they appear on the heat map in Figure 3.

Supplemental Table 3: DEGs induced by developmental AHR activation in resting and responding CD4^{+} T cells that are involved in differentiation pathways

	TCDD resting vs. Vehicle resting		TCDD responding vs. Vehicle responding	
**Differentiation Genes**	**Log_2 Fold Change**	**Adjusted p-value**	**Log_2 Fold Change**	**Adjusted p-value**
Rfng	1.539	0.030	-0.183	0.846
Akap5	1.831	0.048	-0.823	NA
Dvl1	1.366	0.023	0.008	0.995
Il17ra	1.009	0.040	-0.284	0.593
Csnk1g1	0.945	0.097	-1.013	0.019
Hdac8	1.555	0.082	-1.418	0.043
Akt1	1.011	0.010	-0.617	0.082
Smpd4	1.112	0.050	-0.581	0.267
Csnk1d	1.019	0.064	-1.309	0.002
Tyk2	0.942	0.189	-1.046	0.044
Ap1g2	0.255	0.815	-1.553	0.001
Ets1	0.358	0.533	-1.317	0.000
Rps6kb2	1.141	0.097	-1.195	0.023
Inpp5b	0.568	0.355	-1.176	0.002
Numa1	0.410	0.442	-1.089	0.000
Stat6	0.270	0.732	-1.021	0.007
Notch1	0.841	0.215	-1.860	0.000
Shc1	0.520	0.512	-1.080	0.019
Irf7	0.102	0.944	-1.314	0.007
Psap	0.504	0.471	-1.037	0.012
Il21r	0.586	0.396	-1.707	0.000
Il2rg	0.408	0.354	-1.191	0.000
Itpr3	0.307	0.728	-1.188	0.005
Madd	0.440	0.305	-1.077	0.000
Mef2d	0.581	0.351	-1.198	0.002
Smurf1	0.186	0.847	-1.156	0.003
Hdac4	0.665	0.310	-1.005	0.020
Il4ra	0.662	0.333	-1.161	0.008
Mknk1	0.854	0.166	-1.265	0.003
Tsc1	0.842	0.110	-1.025	0.009
Ntrk3	-0.095	0.968	-2.004	0.009
Gna11	0.602	0.661	-1.589	0.027
Gnpda1	0.365	0.744	-1.243	0.021

CD3e	-0.122	0.870	-1.220	0.000
Trp53	-0.036	0.975	-1.764	0.000
Ppox	0.152	0.903	-1.428	0.002
Socs7	-0.011	0.996	-1.320	0.036
Daxx	-0.125	0.925	-1.082	0.029
Tnfrsf1a	0.175	0.899	-1.571	0.002
Il27ra	0.299	0.744	-1.431	0.001
Ncstn	0.540	0.484	-1.776	0.000
Cdc25b	0.344	0.686	-1.131	0.007
Il2rb	0.727	0.229	-1.659	0.000
Pla2g6	0.481	0.736	-1.379	0.053
Hdac10	0.287	0.830	-1.192	0.038
Inpp5k	0.104	0.916	-1.079	0.002
Dtx3	0.453	0.692	-1.566	0.005
Itgb2	0.524	0.364	-1.275	0.000
Rras2	-0.316	0.826	1.378	0.026
Smad7	-0.551	0.534	1.107	0.030
Klrd1	-0.114	0.951	1.558	0.012
Smad1	-0.336	0.795	1.383	0.018
Orai1	0.858	0.167	1.354	0.002
Tob1	0.445	0.691	1.457	0.009
Hmgn1	-0.685	0.402	1.382	0.005
Ppp2cb	-0.213	0.877	1.184	0.033
Cd3d	-0.349	0.500	1.157	0.000
Ndufa13	-0.696	0.161	1.305	0.000
Psenen	-0.754	0.164	1.077	0.005
Calm1	-0.358	0.329	0.707	0.002
Cycs	-0.486	0.366	1.020	0.002
Rheb	-0.408	0.678	1.272	0.009
Arf6	-0.021	0.985	1.011	0.000
Pdia3	-0.264	0.716	1.607	0.000
Casp4	1.174	0.000	0.650	0.029
Bad	-1.173	0.044	0.421	0.471
Mef2c	-2.998	0.000	-0.143	0.898
Cxcr3	-4.080	0.000	-0.887	0.006
Plcb4	-2.872	0.000	-0.471	0.525
Il21	-2.271	0.002	-0.392	0.658
Ccnb1	-2.965	0.000	0.074	0.933
Gna15	-2.324	0.001	0.042	0.968
Bmp7	-1.930	0.030	-0.116	0.927
Il1r1	-2.373	0.002	-0.451	0.607

Cybb	-3.760	0.000	-0.679	0.302
Irf4	-2.088	0.000	-0.314	0.681
Rhoc	-2.029	0.027	0.560	NA
Bcl2a1b	-0.816	0.342	1.327	0.013
Ccl4	-2.269	0.002	1.131	0.063
Adrb2	-2.139	0.000	-1.174	0.004
Hdac2	-1.246	0.038	0.668	0.225
Ifit3	-1.611	0.053	-0.865	0.258
Map2k6	-2.157	0.009	-1.011	NA

Supplemental Table 3: Developmental AHR activation changes expression of genes related to differentiation. Developmental exposure to TCDD impacts expression of genes related to differentiation in both resting and responding $CD4^+$ T cells. The log_2 fold change and adjusted p-value for each gene is listed. An increase in fold change indicates developmental AHR activation increases expression of that gene in $CD4^+$ T cells. Genes were ordered using unsupervised clustering by row, are listed from top to bottom in the order that they appear on the heat map in Figure 4.

Supplemental Table 4: DEGs that are involved in metabolism related pathways in resting and responding CD4^{+} T cells

	TCDD resting vs. Vehicle resting		TCDD responding vs. Vehicle responding	
Metabolism Genes	Log2 Fold Change	Adjusted p-value	Log2 Fold Change	Adjusted p-value
Ndufa1	-0.323	0.684	1.260	0.001
Ndufa11	-0.884	0.091	1.482	0.000
Ndufa12	-0.358	0.636	1.044	0.008
Ndufa13	-0.696	0.161	1.305	0.000
Ndufa2	-0.752	0.165	1.475	0.000
Ndufa3	0.016	0.988	1.285	0.000
Ndufa4	-0.268	0.693	1.190	0.000
Ndufa5	-0.421	0.508	1.334	0.000
Ndufa6	-0.570	0.256	1.364	0.000
Ndufa7	-0.238	0.783	1.318	0.000
Ndufab1	-0.827	0.421	1.451	0.021
Ndufb11	-0.504	0.442	1.195	0.002
Ndufb2	0.233	0.887	1.482	0.022
Ndufb3	-0.270	0.714	1.140	0.001
Ndufb4	0.025	0.989	1.271	0.004
Ndufb5	-0.514	0.305	1.220	0.000
Ndufb6	-0.224	0.810	1.019	0.011
Ndufb7	-0.632	0.222	1.216	0.000
Ndufb9	-0.696	0.257	1.206	0.003
Ndufs4	-0.348	0.556	1.141	0.000
Ndufs6	-0.406	0.311	1.192	0.000
Ndufs8	-0.245	0.718	1.095	0.001
Ndufv3	-0.129	0.871	1.170	0.000
Cyc1	-0.940	0.133	1.215	0.007
Uqcr10	-0.227	0.769	1.564	0.000
Uqcr11	-0.552	0.353	1.422	0.000
Uqcrb	-0.361	0.572	1.230	0.000
Uqcrc1	0.186	0.827	-1.075	0.002
Uqcrfs1	-0.092	0.949	1.280	0.007
Uqcrq	-0.277	0.746	1.459	0.000
Cox6b1	-0.504	0.302	1.359	0.000
Cox6c	-0.373	0.593	1.466	0.000

Cox7b	0.058	0.966	1.423	0.000
Cox7a2	-0.066	0.955	1.616	0.000
Cox8a	-1.198	0.013	0.886	0.033
Atp5d	-1.327	0.018	0.423	0.483
Atp5e	-0.268	0.751	1.620	0.000
Atp5g1	-0.640	0.128	1.187	0.000
Atp5h	-0.365	0.532	1.196	0.000
Atp5j	-0.302	0.553	1.226	0.000
Atp5j2	-0.459	0.445	1.240	0.000
Atp5l	-0.198	0.878	1.396	0.005
Atp5o	-0.530	0.273	1.137	0.000
Aco2	0.696	0.338	-1.317	0.004
Cycs	-0.486	0.366	1.020	0.002
Fis1	-0.746	0.121	1.071	0.002
Ncstn	0.540	0.484	-1.776	0.000
Ogdh	0.333	0.652	-1.276	0.000
Psenen	-0.754	0.164	1.077	0.005
Ucp2	-0.013	0.994	-1.009	0.002

Supplemental Table 4: Developmental AHR activation changes expression of genes related to the electron transport chain. Developmental exposure to TCDD impacts expression of genes involved in the electron transport chain. The $\log_2$ fold change and adjusted p-value for each gene is listed. An increase in fold change indicates developmental AHR activation increases expression of that gene in $CD4^+$ T cells. Genes were grouped according to which ETC complex they are part of, and genes are listed from top to bottom in the order that they appear on the heat map in Figure 5.
